# Gastric Ulcer Secondary to Left Gastric Artery Thrombosis

**DOI:** 10.7759/cureus.45093

**Published:** 2023-09-12

**Authors:** Aidan Farrell, Harshavardhan Sanekommu, Pranav N Shah

**Affiliations:** 1 Internal Medicine, Hackensack Meridian School of Medicine, Nutley, USA; 2 Internal Medicine, Jersey Shore University Medical Center, Neptune City, USA; 3 Radiology, Jersey Shore University Medical Center, Neptune City, USA

**Keywords:** epigastric pain, thrombophilia, left gastric artery, gastrointestinal computed tomography, ischemic gastric ulcer, peptic ulcer disease

## Abstract

Peptic ulcer disease (PUD) is a well-known and commonly encountered gastrointestinal (GI) pathology. *Helicobacter pylori* and nonsteroidal anti-inflammatory drug (NSAID) use are the cause of the majority of PUD cases, although other rare etiologies may be encountered. PUD is confirmed by endoscopic visualization of gastric ulcers, with radiographic imaging being less impactful in diagnosis. In this paper, we present a middle-aged patient who presented with PUD caused by thrombotic occlusion of the left gastric artery (LGA), with her diagnosis being made with computed tomography (CT) imaging prior to endoscopy. This case emphasizes the importance of radiographic imaging in the undifferentiated patient, as well as the unique role radiologists play in both discovering diagnoses and their etiologies.

## Introduction

Peptic ulcer disease (PUD) is a relatively common disease, which affects nearly 90 million people worldwide [1] and is a contributing cause of death in over 250,000 people [2]. Nearly 80%-90% of gastric ulcers are formed as a result of *Helicobacter pylori* infection and/or nonsteroidal anti-inflammatory drug (NSAID) use [3]. It is thought that the development of gastric ulcers is related to alterations in gastric acid secretion and mucosal defense, with external irritants such as NSAIDs and cigarette smoke playing an important role in the pathogenesis of the disease [4]. These alterations in the gastric environment lead to the classic symptom of PUD, which is epigastric pain that is typically described as burning, gnawing, or stabbing that is worse in the morning [4]. Some of the lesser common causes of PUD include malignancy, ischemic disease, and chemotherapy. We report a rare cause of chronic PUD secondary to idiopathic thrombosis of the left gastric artery (LGA) diagnosed by computed tomography (CT) of the abdomen.

## Case presentation

A 61-year-old patient with a past medical history of gastroesophageal reflux disease (GERD) and hypertension presented to the emergency department for a five-day history of severe epigastric pain with associated nausea, vomiting, and diarrhea. The patient has chronic epigastric pain that she attributed to her reflux, which she had an endoscopy for many years ago. Additionally, she has no noted history of recent screening colonoscopy. She describes this pain, which she presented with, as more severe and cramping. The patient thought she had food poisoning, so she tried simethicone and calcium carbonate at home, which did not relieve her symptoms. She denied any intake of nonsteroidal anti-inflammatory drugs (NSAIDs). On arrival, the patient was hemodynamically stable. Her physical examination revealed a patient who did not look ill or in acute distress. Abdominal examination revealed a non-distended abdomen without tenderness upon palpation. All other systems examined revealed no abnormalities. Significant laboratory values were as follows: white blood cell count of 13.2 mm^3^ (reference range: 4,500-11,000 mm^3^), alkaline phosphatase of 109 U/L (reference range: 25-100 U/L), and a lipase of 63 U/L (reference range: 13-60 U/L). Total bilirubin, troponin, and lactate were within normal limits. Her electrocardiogram (EKG) showed normal sinus rhythm.

**Figure 1:**
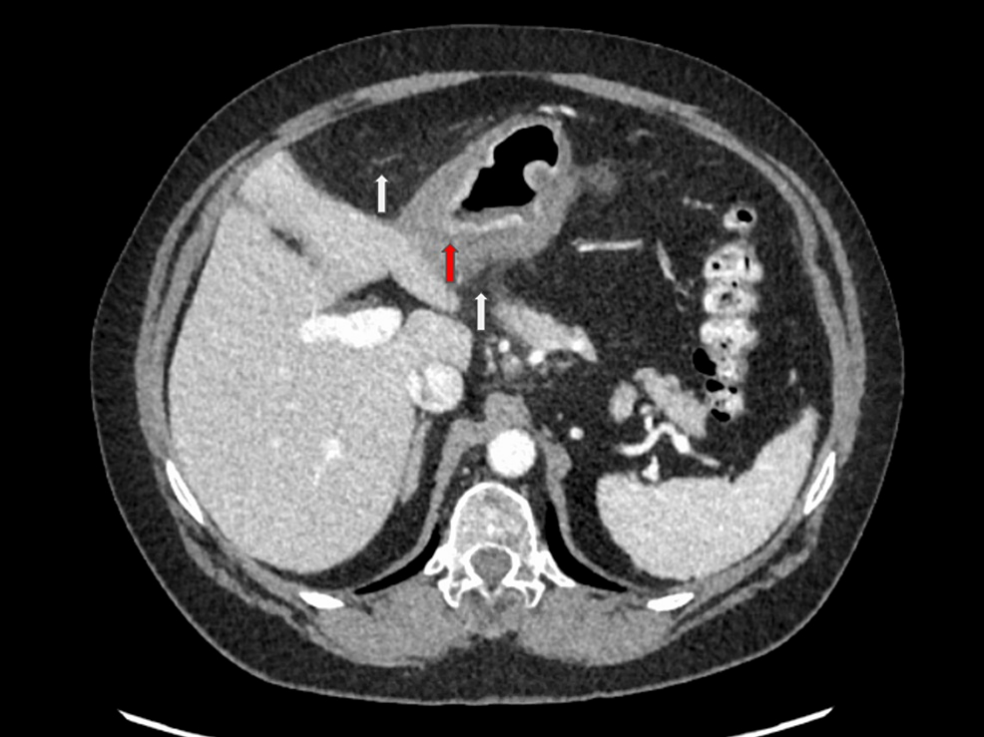
Axial CT of the abdomen and pelvis with contrast: perigastric fat stranding (white arrows) and concentric gastric wall thickening (red arrow) are both present as a result of inflammation. CT: computed tomography

**Figure 2:**
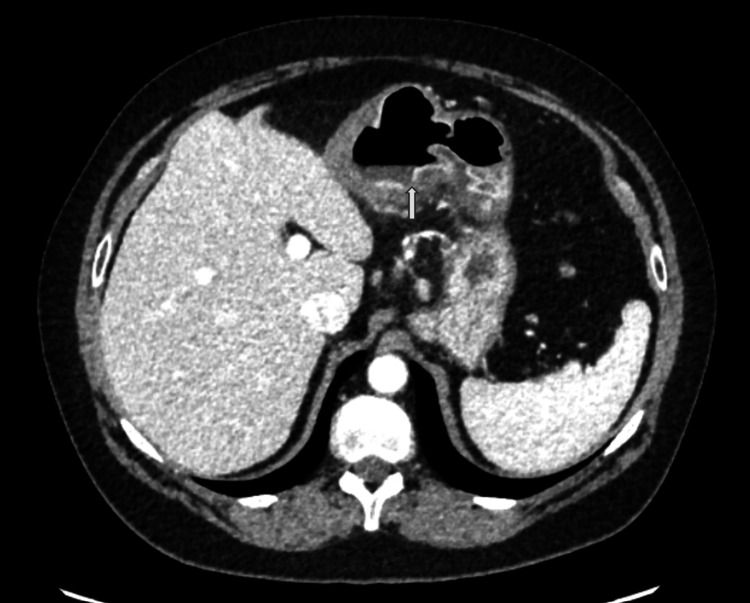
Axial CT of the abdomen and pelvis with contrast: a low-density discontinuity in the mucosa of the gastric antrum is seen (white arrow), which is suggestive of a gastric ulcer in this patient’s presentation. CT: computed tomography

**Figure 3:**
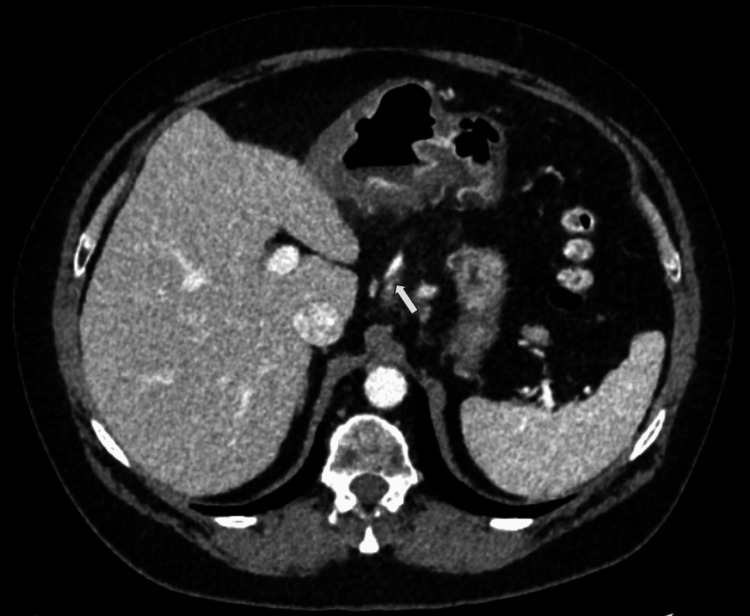
Axial CT of the abdomen and pelvis with contrast shows proximal subtotal occlusion (white arrow) and distal enhancement of the left gastric artery, which suggests high-grade, flow-limiting thrombotic occlusion. CT: computed tomography

Due to her acute-on-chronic presentation of severe abdominal pain, computed tomography (CT) of the abdomen and pelvis with contrast was performed. CT showed concentric mucosal thickening in the antrum of the stomach with adjacent mesenteric fat stranding extending to the gastrohepatic ligament and below the stomach (Figure 1). Discontinuity of the gastric mucosa was evident on the posterior wall of the antrum, raising suspicion of a gastric ulcer (Figure 2). There was no indication of bowel perforation and no intra-abdominal free air was present. Another key imaging finding was a proximal filling defect and distal enhancement of the left gastric artery (LGA), which was thought to be caused by an incomplete thrombotic occlusion of the vessel (Figure 3). All other vessels were patent, and there was no evidence of atherosclerotic disease in the patient. Ischemic-related pathology prompted the patient’s admission.

The patient subsequently underwent esophagogastroduodenoscopy (EGD) with biopsy. EGD was evident for large gastric ulcers with associated clots. Associated biopsies showed reactive foveolar hyperplasia, reactive epithelial changes, and focally active chronic gastritis. These biopsies ruled out *H. pylori* infection and malignancy. Hypercoagulable workup was also unremarkable. She was started on pantoprazole twice daily and apixaban. The patient’s diet was slowly advanced, and once she was able to tolerate a regular diet, she was subsequently discharged after an uneventful five-day hospital stay.

## Discussion

The majority of PUD can be attributed to *Helicobacter pylori* infection and/or NSAID use [3]. Even with knowledge of the most frequent causes, PUD is still a major cause of complications and mortality in an aging population with comorbidities [4]. Less common etiologies include chemotherapy, gastric outlet obstruction, cytomegalovirus, and malignancy [4]. In this paper, we present a patient who developed a gastric ulcer from ischemic causes related to an idiopathic left gastric artery (LGA) thrombus. The workup for suspected PUD begins with testing for *H. pylori* colonization through antibody testing, urea breath testing, and stool antigen testing [4]. The preferred invasive method of PUD evaluation is endoscopy, which allows for the visualization and biopsy of the mucosa. Once PUD is confirmed, medical therapy is the preferred management. This can be the eradication of* H. pylori* through triple therapy of an antisecretory drug, clarithromycin, and amoxicillin, as well as the discontinuation of inciting agents such as NSAIDs and steroids [4]. After medical treatment, patients usually undergo repeat endoscopy after 8-12 weeks to assess for the resolution of the ulcer. Incomplete healing after 12 weeks of medical therapy raises suspicion for malignancy, failed *H. pylori* eradication, and mesenteric ischemia, which would all need further evaluation [4]. In select cases of medical treatment failure or nonadherence, surgery can also be considered.

This case was particularly unique as the patient’s peptic ulcer was not explained by *H. pylori* colonization or chronic NSAID use. Rather, this patient’s ulcer was most likely related to an incomplete thrombotic occlusion of the left gastric artery. The cause of this patient’s ulcer was discovered through CT imaging, which plays a lesser role in the diagnosis of typical cases of PUD. This case shows that on rare occasions, CT may not only diagnose PUD but also its’ etiology.

The images presented demonstrate the left gastric artery (LGA), with proximal subtotal occlusion and distal enhancement, suggesting high-grade, flow-limiting occlusion. Associated with gastric wall thickening, perigastric fat stranding, and discontinuity of the mucosal enhancement, the diagnosis of acute PUD secondary to vascular occlusion can be made. As in other components of the gastrointestinal (GI) tract (small and large bowel), acute vascular occlusion will lead initially to reversible ischemia and then infarct (irreversible) of the supplied tissue, in the absence of secondary sources of perfusion. Fortunately, the gastric body and antrum are richly supplied not only by the LGA but also by the right gastric artery (RGA), gastroduodenal artery (GDA), and gastroepiploic artery (GEA). It is for this fortuitous reason that the damage from acute LGA occlusion results in only a mucosal ulcer (local infarct of a small mucosal focus) rather than a transmural infarct. The vascular collateral arcade described above limits further damage. The LGA arises as the smallest branch of the celiac axis in the vast majority of patients, supplying the distal esophagus and lesser curvature of the body of the stomach. In this case, ulcers of the lesser curvature of the gastric body are demonstrated as focal interruptions in the normal gastric mucosal enhancement and are directly within the flow distribution of the LGA. The LGA thrombosis is characterized by a sudden, abrupt absence of enhancement of the vessel with low-density thrombus filling the lumen. Whether this is due to embolic phenomenon (possible cardiac or aorta source), severe atherosclerosis (unusual location for atherosclerotic disease), or inherent vasculitis (rare cause of LGA occlusion) cannot be ascertained on the CT scan, but a reasonable conclusion would be acute embolus superimposed on the inherently abnormal vessel due to atherosclerosis or vasculitis.

Due to the patient’s unique case of PUD caused by thrombotic occlusion in the absence of other vascular disease, her further workup and treatment deviated from what is described above. The patient was started on a heparin drip, with her abdominal pain significantly improving over the course of four days. Gastroenterology performed an EGD, which confirmed the presence of multiple large non-bleeding ulcers. Mucosal biopsy ruled out H. pylori colonization and malignancy. Additionally, hematology/oncology was consulted, due to concerns of thrombophilia. Hematology/oncology was especially concerned with antiphospholipid antibody syndrome. Their laboratory workup included factor V Leiden, protein C, protein S, prothrombin gene analysis, antithrombin III, anticardiolipin antibody, and beta-2 glycoprotein antibody, which all showed no abnormalities. The patient was discharged on oral anticoagulation after improvement of her abdominal pain and has not returned to our hospital with further complications or disease recurrence.

## Conclusions

To conclude, PUD is commonly encountered worldwide and is associated with significant complications and mortality, particularly in patients with other comorbidities. Usually, PUD is caused by *H. pylori* colonization or the use of gastric irritants such as NSAIDs, with the diagnosis being confirmed endoscopically. Treatment in these cases includes the eradication of *H. pylori* with triple therapy or discontinuation of gastric irritants depending on etiology. In rare instances, CT imaging can play a crucial role in confirming the diagnosis and etiology of PUD, as seen in this case of PUD caused by thrombosis of the left gastric artery.
